# An application of the extended parallel process model to protective behaviors against COVID-19 in South Korea

**DOI:** 10.1371/journal.pone.0261132

**Published:** 2022-03-08

**Authors:** Hyejung Yoon, Myoungsoon You, Changwoo Shon

**Affiliations:** 1 Department of Urban Society Research, The Seoul Institute, Seoul, Korea; 2 Department of Public Health Science, Graduate School of Public Health, Seoul National University, Seoul, Korea; 3 Department of Public Health Science, Graduate School of Public Health, Inje University, Busan, Korea; University of Gondar, ETHIOPIA

## Abstract

This study applied the extended parallel process model (EPPM) to investigate the factors affecting people’s preventive behaviors against COVID-19, and thereby, draw relevant policy implications for current and future other epidemics. The EPPM was used to examine the danger control and fear control responses, along with the separate effects of their sub-factors (perceived susceptibility, perceived severity, response efficacy, and self-efficacy) on personal hygiene behaviors, social distancing measures, and fatalism. In total, data from an online survey of 813 adults were analyzed. The results of multiple regression analysis showed a strong effect of self-efficacy on danger control (*ß* = 0.23 for personal hygiene behaviors, *β* = 0.26 for social distancing) and fear control responses (*ß* = -0.13 for fatalism). However, based on the type of control response, the effect of perceived susceptibility and perceived severity, which were the main factors in threat appraisal, was insignificant or marginally significant. Further, a higher perceived severity was associated with higher fatalism in the fear control response (*ß* = 0.09). Those who were currently employed performed fewer social distancing measures compared to those who did not (*ß* = -0.11), whereas there was no difference in personal hygiene behaviors. These results suggest that risk communication in emerging infectious disease crises should provide customized information on people who are hard to comply with social distancing. Besides delivering the message of self-efficacy, policies should be implemented to create a social environment in which individuals can practice social distancing without constraints.

## Introduction

More than two years has passed since the COVID-19 pandemic has shocked the world. As of February 4, 2022, the World Health Organization had reported 386,548,962 confirmed cases of COVID-19 in 223 countries, including 5,705,754 deaths [1]. The lack of scientific and systematic knowledge about COVID-19 along with the rapid transmission of the disease during the early stages of the pandemic caused tremendous fear and shocked people globally [2, 3]. To prevent the indiscriminate transmission of the novel infection, movement between countries and cities was blocked, and in some cases, even within the city. The daily lives of people were limited due to social distancing policies [4, 5]. The modern society of the 21^st^ century, which has enjoyed the benefits of cutting-edge science and technology, experienced a novel and persistent shock.

When it comes to public health emergencies such as infectious diseases, compliance with the public health guidelines related to preventive actions by individuals is critical [6, 7]. In particular, it is crucial to understand the psychological processes related to an individual’s voluntary adherence and protective measures to successfully respond to a new infectious disease [8]. Public adherence to government guidelines varies from individual to individual; therefore, even in threatening public health crises such as COVID-19, some people do not engage in protective measures. Hence, it is very important to understand what drives people to follow precautions, not only in the context of the COVID-19 pandemic but also in past outbreaks and for future public health emergencies.

South Korea (hereinafter Korea) also implemented social distancing measures to prevent the transmission of COVID-19. However, unlike other countries that imposed compulsory and strict control measures, such as city or border lockdowns, Korea adhered to its population’s active and voluntary participation in social distancing measures. Through extensive and rigorous contact tracing and early detection of confirmed cases via rapid mass testing, Korea was able to prevent the transmission of infection without any lockdown measures [9, 10]. Under such open and flexible control measures, it is even more essential that people voluntarily participate in the government’s social distancing guidelines.

In an unknown and newly emerging crisis situation, the general public’s affective and emotional responses are heightened [11]. Furthermore, how people perceive a threat is closely related to the implementation of recommended preventive behaviors in such a crisis situation. Recent risk communication research emphasizes that individuals’ attitudes based on emotions and feelings should be noted to understand the public’s risk perception of the hazard [12, 13]. The extended parallel process model (EPPM) is a theoretical model in psychosocial and behavioral sciences that explains when and why people follow health recommendations. The EPPM, an integrated and comprehensive model of affective and cognitive processes, describes the behavioral change process as the relationship between the fear of a health message or health risk situation, and the individual’s sense of efficacy in coping strategies [14, 15]. Similar to the EPPM, behavior change models, such as the health belief model (HBM) [16] and protection motivation theory (PMT) [17], explain that an individual’s perceived vulnerability and efficacy are important factors influencing their behavioral change. However, in the case of EPPM, affective and emotional responses such as anxiety and fear are assumed in the theoretical model itself. Therefore, this study decided that EPPM is more suitable in a crisis such as the COVID-19 pandemic. The EPPM focuses on two components: perceived threat and perceived efficacy [15]. The perceived threat involves perceived severity and susceptibility; perceived efficacy consists of self-efficacy and response efficacy. Perceived susceptibility refers to the likelihood of being influenced by a threat [18]. Perceived severity refers to the extent to which one believes that a threat is serious [18]. Perceived efficacy relates to the effectiveness and feasibility of a recommended preventive action [18]. Response efficacy is the belief about the effectiveness of the recommended actions, while self-efficacy refers to one’s beliefs that one can follow the recommendations [18].

In fear appeal models such as the PMT and EPPM, fear is a factor that triggers an individual’s behavioral change to the recommended action. It is an important attribute of infectious disease crises compared to other crises [11]. According to the EPPM [15], the danger control response, which is the most ideal state for change to the recommended health behavior, occurs when the perceived threat is high and the individual’s sense of efficacy is greater than the threat. Efficacy appraisal is a danger control process; perceived efficacy plays a critical role in this process. When perceived efficacy is lower than the perceived threat, the individual exhibits the fear control response, which is a defensive response to control the fear itself. Threat appraisal is described as a fear control process; the perceived threat is a key factor in determining this process [18, 19]. Perceived threat determines the intensity or magnitude of the response to the threat, whereas perceived efficacy determines the characteristics of the response, which is either fear control or danger control [20, 21].

The EPPM explains that danger control and fear control are mutually exclusive responses based on an individual’s threat and efficacy appraisal. Fear control, the process of reducing fear itself, appears as defensive avoidance, message derogation, and perceived manipulation [22]. In relation to infectious disease, people may take actions such as thinking that the government exaggerates the threat (message derogation), or turning off broadcasts when related messages are displayed because they do not want to think about the infection or the virus itself (defensive avoidance) [23, 24]. Fatalism is a reaction that can appear as a fear-control response [22, 25]. Fatalism is the belief that an individual’s actions have no significant effects on important outcomes [26, 27]; it is expressed in the form of predetermination, luck, and/or pessimism [28]. However, few studies have been conducted on fear control responses [22].

According to prior research, fatalism was negatively associated with performing actions to mitigate the transmission of COVID-19 [28, 29]. In addition, the four factors–perceived susceptibility, perceived severity, response efficacy, and self-efficacy–may play distinct roles in explaining the recommended health behavior performance [30, 31]. However, in many studies on the EPPM, especially those related to the COVID-19 pandemic, perceived threat and perceived efficacy were used as the sum or product of each sub-factor [23, 24, 32]. According to Popova [22], who reviewed 29 studies on EPPM, the effects of perceived threat and efficacy were mixed. Studies that have analyzed the factors related to social distancing participation according to the EPPM have typically investigated the danger control response [19, 31, 32] or both the fear control and danger control responses using perceived threat and perceived efficacy in the form of composite measures [23, 24]. Therefore, this study aimed to investigate the relationship between people’s preventive behavior against COVID-19 and individual psychosocial variables during the early stage of the COVID-19 pandemic in South Korea based on the EPPM model.

## Methods

### Design and participants

This study used a cross-sectional survey design via an online platform. The survey was administered by a professional survey company called Hankook Research from April 28 to May 1, 2020. A proportionate quota sampling method was used to recruit the participants, based on age and sex, from all five regions of Seoul to ensure the representativeness of the entire population. The participants were recruited from a total of 460,000 online panels. E-mail and mobile text messages were sent to 3,773 people, and 1,007 people participated in the survey. After participants with incomplete questionnaire data were removed, 813 people (completion rate 80.7%) were included in the final analysis. When the participants visited the online survey site, they were provided with detailed information about the study and were informed that their participation in the survey was voluntary. They were also informed that their response data would only be used for statistical and research purposes that were outlined on the first page of the online survey screen. Only those who chose the “Yes” button on the screen for an online informed consent form were allowed to participate; participants could stop or leave the survey at any time.

The survey had a margin of error of ±3.4% at a 95% confidence interval. A priori power analysis, conducted using the G*Power program (version 3.1.9.7) [33], revealed that this sample was sufficient for valid results to detect small, medium, and large effects. Considering a high power of 0.90, an alpha level of 0.05, a low effect size of f^2^ = 0.02, and a number of 13 predictors, we obtained a total sample size of 776 participants with a critical F of 2.38. This study was approved by the Institutional Review Board of the Seoul Medical Center (No. 2020-04-005), and conducted in accordance with the principles of the Declaration of Helsinki and its amendments.

### Measures

The sociodemographic variables included in the analysis were sex (0 = men, 1 = women), age (in years), education (0 = high school or below, 1 = college or above), monthly household equivalence income (values ranging from 1 (under 1,724 US dollars(USD)) to 4 (5,143 USD or above), and employment status (0 = unemployed, 1 = employed)). People with chronic diseases are highly vulnerable to COVID-19 infection [34]; research suggests that an individual’s health status is associated with performing COVID-19 preventive measures, COVID-19 pandemic fear, and risk perception [35, 36]. Therefore, we asked participants about their health status. Participants were asked whether they had any chronic diseases that were diagnosed by a physician or had received treatment for chronic disease in the last year; they also completed a single-item measure that assessed self-rated health status on a 5-point Likert scale that ranged from “bad” to “excellent.” Responses to the self-rated health status item were recoded as a dummy variable: 0 for “bad health status” and 1 for “good health status.” Nine diseases, including hypertension, diabetes, stroke, cancer, and asthma, were presented following the chronic disease statistics of the National Health Insurance Service. The relevant descriptive statistics are reported in Table 1.

**Table 1:** Descriptive statistics and psychological distress of the participants (*n* = 813).

Characteristics	*n*	*%*
Sex		
Men	377	46.4
Women	436	53.6
Age Groups	*M* = 46.0	*SD* = 14.9
18–29	152	18.7
30–39	149	18.3
40–49	152	18.7
50–59	146	18.0
Over 60’s	214	26.3
Highest level of educational attainment		
High school or below	215	26.5
College and above	598	73.5
Monthly household equivalence income		
< $1,714 USD	161	19.8
$1,714 –$3,427 USD	407	50.1
$3,428 –$ 5,142 USD	106	13.0
≥ $5,143 USD	139	17.1
Employment		
Unemployed	301	37.0
Employed ^b^	512	63.0
Health status		
Bad	385	47.4
Good	428	52.6
Presence of chronic diseases		
No	549	67.5
Yes	264	32.5

USD: US dollar (USD 1 = Korean won 1,166.72 based on the basic exchange rate on 2019); *M*: Mean; *SD*; Standard Deviation.

The protective behaviors against COVID-19 were measured using nine items following the Central Disaster Management Headquarters (CDMH) and Central Disease Control (CDCH) social distancing and prevention recommendations; the aim was to assess how frequently individuals participated in those actions in the past week. Three items measured personal hygiene behaviors (*α* = 0.66, M = 3.67, SD = 0.45) and included: (1) wearing a mask, (2) washing hands, and (3) covering mouth with the sleeve when coughing. The remaining six items assessed social distancing performance (α = 0.82, M = 2.96, SD = 0.67) and included: (1) avoided visiting public places, (2) reduced the use of public transportation, (3) postponed or canceled social events, (4) refrained from going out, (5) stayed at home for three to four days if sick, and (6) kept a 2 m distance from other people. Participants responded to each item on a 4-point Likert-type scale (1 = never, 2 = sometimes, 3 = often, or 4 = always). The items were summed and averaged to derive the performance scores of personal hygiene behaviors and social distancing performance, with higher scores indicating that participants performed more protective behaviors (see Table 2 for more details).

**Table 2:** Mean values, standard deviations, Cronbach’ s alpha, and Pearson correlations between the variables in regressions.

Variables	M (SD)	(1)	(2)	(3)	(4)	(5)	(6)	(7)
(1) Personal Hygiene	3.67 (0.45)	(0.67)						
(2) Social distancing	2.96 (0.67)	0.39***	(0.82)					
(3) Fatalism	3.41 (0.82)	-0.08*	-0.09*	(0.74)				
(4) Perceived susceptibility	2.70 (0.74)	0.01	0.07	0.06	-			
(5) Perceived severity	3.87 (0.82)	0.08*	0.10**	0.08*	0.22***	-		
(6) Response efficacy	3.69 (0.44)	0.34***	0.38***	-0.02	0.05	0.09*	(0.81)	
(7) Self-efficacy	5.04 (1.04)	0.31***	0.39***	-0.14***	-0.05	0.08*	0.33***	(0.77)

*Note*. *n* = 813, Cronbach’s alpha coefficients are in parentheses along the diagonal.

* *p* < 0.05.

** *p* < 0.01.

*** *p* < 0.001.

To measure perceived threat, perceived susceptibility and perceived severity were measured following previous research in Korea on COVID-19 [37]. The items included: “How likely do you think you are to be infected with the coronavirus?” and “If you were infected with the coronavirus, how serious would your health damage be?”. Items were scored on a 5-point scale ranging from very low (score = 1), neither low nor high (score = 3), to very high (score = 5) (see Table 2 for more details).

Response efficacy (*α* = 0.81, M = 3.69, SD = 0.44) was assessed with four items. Participants were asked, “How helpful do you think the following actions are in preventing the spread of COVID-19?”, on a 4-point Likert-type scale (1 = not at all to 4 = extremely) based on the recommended preventive measures: (1) personal precautions (wearing a mask, washing hands, cough etiquette, etc.); (2) avoiding crowded situations (public transportation, multi-use facilities, etc.); (3) avoiding contact with other people (cancellation, absence from meetings, and refraining from going out); and (4) taking three–four days off if you are sick. The four items were summed and averaged on a single scale (see Table 2 for more details).

At the time of the survey, the CDMH and CDCH announced a plan to change to a more flexible social distancing system, “distancing in daily life”. Therefore, we developed four items to measure self-efficacy (*α* = 0.77, M = 5.04, SD = 1.04) of the previous strict social distancing, even if they were changed to distancing in daily life. Participants were asked to what extent they agreed or disagreed with the following four statements: (1) “I will thoroughly practice social distancing rules even if ‘the enhanced social distancing’ shifted to ‘distancing in daily life’,” (2) “It is difficult for me to practice social distancing” (reverse coded in analysis), (3) “Even if I switch to distancing in daily life, I could practice social distancing well,” and (4) “My surrounding and living conditions are equipped to practice social distancing.” Responses ranged from “completely disagree” (score = 1), “neutral” (score = 4), to “completely agree” (score = 7). The four items were summed and averaged on a single scale (see Table 2 for more details).

Fatalism (*α* = 0.74, M = 3.41, SD = 0.82) was assessed using three items on a scale of 1 (strongly disagree) to 5 (strongly agree). The items included: (1) “No matter how careful I am, I cannot prevent the infection itself”; (2) “Whether I get infected or not is a matter of luck”; and (3) “What will happen is about to happen.” The three items were summed and averaged on a single scale (see Table 2 for more details).

All measures are listed in S1 File.

### Data analysis

All analyses were conducted using SAS version 9.4 and statistical significance was set at *p* < 0.05. Descriptive statistics were used to explore the participants’ general characteristics. The normality of the distribution of univariate variables was assessed based on skewness and kurtosis values. All variables presented acceptable values for the proposed threshold of skewness (within ± 2.0) and kurtosis (within ± 7.0) [38], suggesting a normal distribution for the variables. Multiple regression analysis was conducted for each dependent variable (personal hygiene behavior and social distancing measures for danger control response, and fatalism for fear control response). The predictor variables were sex (men versus women), age (continuous), education (high school graduate and below versus college or above), monthly household equivalence income (under 1,714 USD as reference), employment status (unemployed versus employed), subjective health status (bad versus good), presence of a chronic condition (no versus yes), perceived susceptibility, perceived severity, self-efficacy, and response efficacy. The absence of multicollinearity between the variables was ensured by correlational analyses: all correlations were 0.39 or below (Table 2). We also examined variance inflation factor statistics to evaluate multicollinearity [38]; the highest individual variance factor was for the income variable at 1.87.

## Results

**Fig 1:**
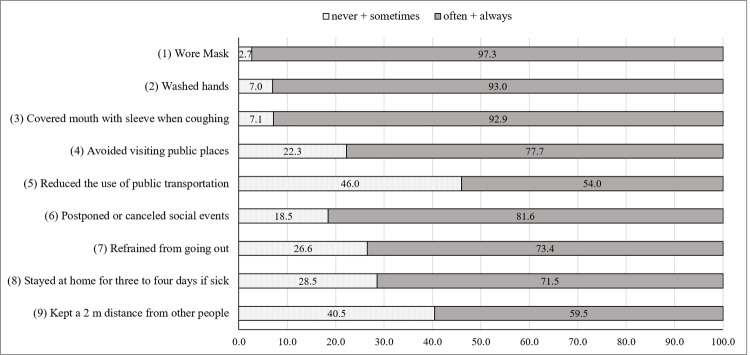
Frequency of protective behaviors against COVID-19.

Fig 1 shows a basic frequency analysis of the nine precautionary behaviors for COVID-19. Responses that used a 4-point Likert-type scale were divided into two groups: Group 1 included “never” or “sometimes,” and Group 2 included “often” or “always.” Personal hygiene behaviors (i.e., wearing a mask, washing hands, and covering mouth with the sleeve when coughing) showed a high degree of compliance. Notably, more than 90% of the participants responded that they often or always practiced these preventive behaviors. For social distancing, “postponed or canceled social events” was the most practiced measure (81.6% answered often or always), followed by “avoided visiting public places” (77.7%). However, “reduced the use of public transportation” and “kept a 2 m distance from other people” showed a relatively low rate of compliance.

### Fatalism—Fear control response

The model for fatalism as a fear control response was significant, with *F* (13, 799) = 2.90, *p* < 0.001; however, the variables explained only 3.0% of the variance of fatalism. Among them, only perceived severity and self-efficacy showed a significant association. According to the EPPM, fear control response occurred when a perceived threat was high, but the efficacy was low. This meant that individuals let their emotions take over and concentrated on the “fear.” The results showed that people with high perceived severity had high levels of fear control (*ß* = 0.085, *p* < 0.05), but people with higher levels of self-efficacy had lower scores for the fear control response (*ß* = -0.126, *p* = 0.001) (see Table 3 for more details).

**Table 3:** Multiple regression analysis with the variables predicting fatalism.

	Fatalism			
Variables	b (SE)	ß	*t*	*P*
Intercept	3.633 (0.305)	0.000	11.93	<0.001
Sex (ref. Men)	-0.03 (0.06)	-0.018	-0.49	0.625
Age group (ref. 20s)	-0.003 (0.002)	-0.056	-1.34	0.182
Education (ref. Under high school)	0.11 (0.069)	0.059	1.61	0.109
Presence of chronic condition (ref. No)	-0.107 (0.071)	-0.061	-1.5	0.133
Subjective health status (ref. Bad)	-0.096 (0.061)	-0.058	-1.58	0.114
Monthly household equivalence income (ref. < $ 1,714 USD)				
$1,714 –$3,427 USD	-0.102 (0.078)	-0.062	-1.32	0.188
$3,428 –$ 5,142 USD	-0.161 (0.104)	-0.066	-1.54	0.125
≥ $5,143 USD	-0.043 (0.098)	-0.020	-0.44	0.662
Employment (ref. Unemployed)	-0.028 (0.065)	-0.017	-0.43	0.664
Perceived susceptibility	0.038 (0.039)	0.035	0.97	0.333
Perceived severity	0.085 (0.036)	0.085	2.33	0.02
Response efficacy	0.030 (0.07)	0.016	0.43	0.669
Self-efficacy	-0.100 (0.03)	-0.126	-3.3	0.001

USD: US dollar (USD 1 = Korean won 1,166.72 based on the basic exchange rate on 2019).

### Personal hygiene behaviors—Danger control response

The model for personal hygiene behaviors as a danger control response was significant, with *F* (13, 799) = 15.57, *p* < 0.001, and adjusted *R*^*2*^ = 0.19. Women (*ß* = 0.118, *p* = 0.001), and people with high levels of self-efficacy (*ß* = 0.232, *p* < 0.001) and response efficacy (*ß* = 0.228, *p* < 0.001) engaged in more personal hygiene behaviors. Those who had one or more chronic diseases participated lesser in the performance of personal hygiene activities compared with those without a chronic disease (*ß* = -0.092, *p* < 0.05) (Table 4).

**Table 4:** Multiple regression analysis predicting the danger control response- personal hygiene behaviors.

	Model 1				Model 2			
Variables	b (SE)	ß	*t*	*p*	b (SE)	ß	*t*	*p*
Intercept	2.174 (0.152)	0.000	14.33	<0.001	2.287 (0.164)	0.000	13.91	<0.001
Sex (ref. Men)	0.106 (0.03)	0.118	3.53	<0.001	0.105 (0.03)	0.117	3.5	0.001
Age group (ref. 20s)	-0.001 (0.001)	-0.034	-0.88	0.379	-0.001 (0.001)	-0.037	-0.96	0.335
Education (ref. Under high school)	0.046 (0.034)	0.045	1.35	0.179	0.049 (0.034)	0.049	1.45	0.149
Presence of chronic condition (ref. No)	-0.088 (0.035)	-0.092	-2.49	0.013	-0.091 (0.035)	-0.096	-2.58	0.010
Subjective health status (ref. Bad)	0.048 (0.03)	0.053	1.58	0.115	0.045 (0.03)	0.050	1.48	0.140
Monthly household equivalence income (ref. < $ 1,714 USD)								
$1,714 –$3,427 USD	-0.005 (0.039)	-0.006	-0.13	0.894	-0.008 (0.039)	-0.009	-0.22	0.829
$3,428 –$ 5,142 USD	-0.005 (0.052)	-0.004	-0.09	0.928	-0.01 (0.052)	-0.007	-0.19	0.851
≥ $5,143 USD	0.03 (0.049)	0.025	0.61	0.54	0.029 (0.049)	0.024	0.59	0.558
Employment (ref. Unemployed)	-0.029 (0.032)	-0.032	-0.91	0.361	-0.03 (0.032)	-0.033	-0.94	0.347
Perceived susceptibility	-0.001 (0.02)	-0.002	-0.05	0.958	0.000 (0.02)	0.000	0.01	0.993
Perceived severity	0.029 (0.018)	0.054	1.61	0.108	0.032 (0.018)	0.059	1.75	0.08
Response efficacy	0.231 (0.035)	0.228	6.59	<0.001	0.232 (0.035)	0.229	6.63	<0.001
Self-efficacy	0.100 (0.015)	0.232	6.65	<0.001	0.097 (0.015)	0.225	6.4	<0.001
Fatalism					-0.031 (0.018)	-0.057	-1.78	0.076

USD: US dollar (USD 1 = Korean won 1,166.72 based on the basic exchange rate on 2019).

Further, the effect of fatalism on the performance of personal hygiene behaviors was analyzed as follows: first, demographic, health-related, and EPPM variables were controlled; and second, fatalism was subsequently added to the model (Table 4). Fatalism was negatively associated with personal hygiene behaviors; however, this association was marginally significant (*ß* = -0.057, *p* < 0.1).

### Social distancing—Danger control response

The model for social distancing was significant, with *F* (13, 799) = 21.93, *p* < 0.001, and adjusted *R*^*2*^ = 0.25. Social distancing was positively associated with being older (*β* = 0.112, *p* < 0.01), unemployed (*β* = -0.109, *p* = 0.001), and having better self-efficacy (*β* = 0.264, *p* < 0.001), and response efficacy (*β* = 0.241, *p* < 0.001). Notably, the effect size of self-efficacy was the largest, followed by response efficacy. Sex and perceived susceptibility showed a positive association with undertaking social distancing measures against COVID-19; however, the effects were marginally significant (*β* = 0.058, *p* < 0.10 for sex, and *β* = 0.061, *p* < 0.10 for perceived susceptibility). The effect of perceived severity was not significant (Table 5).

**Table 5:** Multiple regression analysis predicting the danger control responses- social distancing.

	Model 1				Model 2			
Variables	b (SE)	ß	*t*	*p*	b (SE)	ß	*t*	*p*
Intercept	0.223 (0.219)	0.000	1.02	0.309	0.338 (0.238)	0.000	1.42	0.156
Sex (ref. Men)	0.079 (0.043)	0.058	1.82	0.070	0.078 (0.043)	0.058	1.8	0.073
Age group (ref. 20s)	0.005 (0.002)	0.112	3.06	0.002	0.005 (0.002)	0.110	3.0	0.003
Education (ref. Under high school)	0.083 (0.049)	0.055	1.69	0.092	0.087 (0.049)	0.057	1.76	0.079
Presence of chronic condition (ref. No)	0.038 (0.051)	0.027	0.74	0.457	0.035 (0.051)	0.024	0.68	0.498
Subjective health status (ref. Bad)	0.051 (0.044)	0.038	1.16	0.245	0.048 (0.044)	0.035	1.09	0.275
Monthly household equivalence income (ref. < $ 1,714 USD)								
$1,714 –$3,427 USD	-0.046 (0.056)	-0.034	-0.82	0.413	-0.049 (0.056)	-0.036	-0.88	0.381
$3,428 –$ 5,142 USD	-0.004 (0.075)	-0.002	-0.05	0.961	-0.009 (0.075)	-0.004	-0.12	0.907
≥ $5,143 USD	0.014 (0.07)	0.008	0.19	0.847	0.012 (0.07)	0.007	0.17	0.862
Employment (ref. Unemployed)	-0.152 (0.047)	-0.109	-3.27	0.001	-0.153 (0.047)	-0.110	-3.28	0.001
Perceived susceptibility	0.055 (0.028)	0.061	1.93	0.054	0.056 (0.028)	0.062	1.97	0.049
Perceived severity	0.028 (0.026)	0.034	1.05	0.293	0.03 (0.026)	0.037	1.15	0.25
Response efficacy	0.368 (0.051)	0.241	7.27	<0.001	0.369 (0.051)	0.242	7.29	<0.001
Self-efficacy	0.172 (0.022)	0.264	7.88	<0.001	0.169 (0.022)	0.260	7.69	<0.001
Fatalism					-0.032 (0.025)	-0.039	-1.24	0.215

USD: US dollar (USD 1 = Korean won 1,166.72 based on the basic exchange rate on 2019).

Similar to personal hygiene behaviors, the effect of fatalism on performing social distancing was analyzed as follows: First, demographic, health-related, and EPPM variables were controlled; and second, fatalism was subsequently added to the model. Fatalism was negatively associated with social distancing activities; however, this effect was not significant (Table 5).

## Discussion

This study applied the EPPM to understand the factors that affect an individual’s participation in protective behaviors against COVID-19. Nonpharmaceutical interventions, including social distancing measures, are effective and powerful in preventing the transmission of an infection in the event of an emergence of a novel infectious disease, such as COVID-19. Understanding an individual’s perception of both the disease (threats) and the countermeasures against the infection is crucial in persuading the public to follow the recommended activities. This study investigated the effect of public perception of threat and efficacy on fatalism and undertaking protective behavior based on the EPPM framework. This study also demonstrated the applicability of the EPPM framework to preventive behaviors during the COVID-19 pandemic. Importantly, this study’s findings provide necessary information for establishing a public health emergency preparedness system that can cope with the outbreak of another infectious disease in the future.

First, this study’s findings according to respondents’ characteristics are described as follows. Responses to COVID-19 differed demographically. Women undertook more preventive measures against COVID-19 than men, as reported in previous studies [23, 37, 39]. Further, those who were younger (employed) undertook fewer social distancing measures than those who were older (unemployed).

Furthermore, actions differed between those with and without chronic diseases. The former engaged in fewer personal hygiene behaviors to prevent COVID-19 infection compared to those who did not. Due to high susceptibility to infection and the occurrence of complications with mortality, patients with chronic diseases are classified into a high-risk group in an infectious disease crisis; the COVID-19 pandemic is no exception [34, 40]. Therefore, they may reduce going out and take more preventive actions during the COVID-19 pandemic [40, 41]. Reportedly, many such patients do not visit the hospital for regular check-ups due to the risk of COVID-19 infection [42]. This study also found that people with chronic diseases performed better on following social distancing guidelines by reducing going out and avoiding crowded places. As people with chronic diseases went out less during the early stage of the COVID-19 pandemic, it is likely that their performance in personal hygiene practices, such as wearing a mask and washing hands, has decreased.

Next, the findings related to sub-factors of the EPPM are outlined as follows. The effect of perceived threat, which was one of the key factors of the EPPM, was insignificant or marginally significant based on the type of control response. Only perceived susceptibility marginally explained social distancing performance, whereas perceived severity did not significantly explain both personal hygiene behaviors and social distancing. People participated more in personal hygiene activities than in social distancing. Furthermore, even as the employed performed fewer social distancing measures than the unemployed, there was no difference in personal hygiene behaviors. This finding may be related to Korea’s previous experience with the Middle East respiratory syndrome, or MERS, in 2015 [43]. In addition, before the outbreak of COVID-19, there was a severe air pollution problem, such as fine dust; therefore, people were relatively accustomed to wearing a facial mask and washing hands in daily life compared to other countries [43]. For Koreans, personal hygiene behavior to reduce the risk of infection with COVID-19 was not a completely new preventive measure, but a familiar behavior that was routinely performed. Moreover, people were already aware of the effectiveness of these behaviors. Therefore, perhaps perceived efficacy was more related to preventive behavior than an individual’s risk perception about the COVID-19 virus.

In the fear control response, perceived severity was positively associated with a higher level of fatalism. Contrary to the proposition that perceived efficacy was not related to the fear control response [20], this study found that an individual’s self-efficacy was negatively associated with fatalism. This may be related to the measurement of the fear control response as fatalism. We investigated only the fatalism among various fear control responses. Besides fatalism, an individual can control the feeling of fear with actual actions, such as defensive avoidance and message derogation [23, 24]. This suggests that the effect of perceived threat and efficacy in the EPPM may differ based on the type of fear control response. Further investigation is required on this concept.

Furthermore, self-efficacy significantly affected the danger and fear control responses in the EPPM. The higher the self-efficacy, the higher the performance of personal hygiene behaviors and social distancing measures, and lower fatalism toward COVID-19 infection. Response efficacy was also positively associated with a higher level of performance in protective behaviors. These results suggest that it was generally most important to have high levels of self-efficacy to implement the recommended preventive measures. Self-efficacy is known to be a factor that has strong explanatory power for an individual’s health behavior in behavioral and cognitive theories, including HBM [16, 44], PMT [17, 45, 46], and EPPM [7, 39, 47].

An individual’s voluntary participation in social distancing without coercion is very effective and crucial during the transmission of a new infectious disease [6, 7]. Self-efficacy is the perceived ability to perform recommended behaviors, and enables individuals to practice social distancing measures voluntarily and actively. However, even if an individual wants to practice social distancing, sometimes it cannot be implemented due to environmental constraints. “Staying at home for three to four days if sick” is the most representative example. Korea is one of the four members of the Organization for Economic Cooperation and Development that does not guarantee the right to paid sick leave for workers [48, 49]. In the absence of sickness benefits, workers who are unable to work from home have to bear the disadvantages of absence and the associated costs [50]. The mass infection of COVID-19 in a call center in Seoul, which occurred during the first wave of the pandemic, was a representative example of a working environment that made it difficult to practice social distancing, such as “resting for 3–4 days if you are sick” in the absence of sickness benefits [50]. Fortunately, social and political discussions on the introduction of sickness benefits have begun in Korea [51, 52]. This study emphasizes the importance of policies that create social conditions for individuals to practice social distancing in accordance with the government’s recommendations during crises.

This study investigated the distinct effects of each sub-factor of the EPPM: perceived susceptibility, perceived severity, response efficacy, and self-efficacy. However, besides these related factors, many other factors are involved in undertaking protective behaviors against COVID-19. One factor may be involuntary participation. When strong social norms force people to engage in protective behaviors, individuals may act under the normative pressure to follow [8]. The required social distancing measures against COVID-19 and the degree of compliance of the public would vary depending on the culture of each country [19]. Studies have shown that in Asian countries, such as China and Korea, collectivism is relatively dominant and people’s conformity to the group culture is high [19, 53]. Therefore, the results of this study may also reflect cultural characteristics.

This study has some limitations. First, a cross-sectional design with an online survey was used here. Therefore, the results should be interpreted as an association and not as a cause-and-effect relationship. Second, social desirability bias may be present due to self-reported data. The survey was conducted during the first wave of the COVID-19 pandemic in Korea; at that time, it was impossible to conduct a face-to-face survey. Notably, there was no qualitative difference in the responses of sensitive personal information, such as privacy, according to the survey methods [54]. Therefore, the social desirability bias related to the self-reported online survey may not have caused a major problem in influencing the results of this study.

Although many studies have been conducted to analyze the preventive behavior of COVID-19 by applying EPPM in Europe [7], the United States [19], Canada [23], and Asia [19, 21], to the best of our knowledge, this study is the first to investigate social distancing participation factors through the EPPM framework in Korea; specifically, this is evaluated as successfully coping with the spread of COVID-19 [36]. This study demonstrates that EPPM can explain an individual’s behavior in coping with infectious diseases as an emotional and cognitive dynamic process, even in culturally and politically different contexts. Using the EPPM framework, this study provides clear evidence that an individual’s self-efficacy and demographic characteristics are predictors of social distancing during a pandemic crisis.

Finally, based on the results, this study has some recommendations as well. First, risk communication should provide customized information according to the social distancing risk groups, such as men, young people, and workers unable to work from home. Second, the message should emphasize an individual’s self-efficacy: that the individual can fully follow the preventive actions and government recommendations. Lastly, besides delivering the message of self-efficacy, policies should be implemented to create a social environment in which individuals can practice social distancing without constraints.

Even though vaccinations have begun, the COVID-19 pandemic is still ongoing. While the end of the pandemic is uncertain, there is also a risk of another infectious disease outbreak in the near future. Social distancing is a key countermeasure for future infectious disease responses and risk communication management. To prevent infection through social distancing strategies, we should provide people with clear, easy-to-understand, and repetitive information about what to do, as well as why and how to do it [8]. For a sustained effective response during a prolonged infectious disease situation, institutional improvements should be undertaken to enhance the ability and capacity to respond to infectious diseases.

## Supporting information

S1 File Survey questionnaires. (PDF)
