# Supplementary material for: An application of the extended parallel process model to protective behaviors against COVID-19 in South Korea
Source: PLoS One. 2022 Mar 8;17(3):e0261132. doi: 10.1371/journal.pone.0261132 (PMC8903272; doi:10.1371/journal.pone.0261132)
Supplement: S1 File — (PDF) [file pone.0261132.s001.pdf]

Supporting information file 1. Survey questionnaire

The information described below provides the questionnaire used in this study which was developed for this study.

[Perceived threat]

#	Perceived threat of COVID-19 infection	Very low		Neither low nor high		Very high
Q1	What do you think is the possibility of your COVID-19 infection? [***perceived susceptibility***]	1	2	3	4	5
Q2	What do you think will be the severity if COVID-19 infects you? [***perceived severity***]	1	2	3	4	5

[Response efficacy]

#	Response efficacy	Not at all			Extremely
	**How helpful do you think the following actions are in preventing the spread of COVID-19?**				
Q3	Practicing personal precautions such as wearing facial masks, hand hygiene, and cough etiquette	1	2	3	4
Q4	Avoiding crowded situations such as public transportation, multi-use facilities	1	2	3	4
Q5	Avoiding contact with other people such as cancellation, absence from meetings, and refraining from going out	1	2	3	4
Q6	Taking three to four days off if you are sick	1	2	3	4

[Self-efficacy]

#	Self-efficacy	Completely disagree			Neutral			Completely agree
Q7	I will thoroughly practice social distancing rules even if 'the enhanced social distancing' shifted to 'distancing in daily life.'	1	2	3	4	5	6	7
Q8	It is difficult for me to practice social distancing.	1	2	3	4	5	6	7
Q9	Even if I switch to distancing in daily life, I could practice social distancing well.	1	2	3	4	5	6	7
Q10	My surrounding and living conditions are equipped to practice social distancing.	1	2	3	4	5	6	7

[Fatalism]

#	Fatalism	Strongly disagree		Neutral		Strongly agree
Q11	No matter how careful I am, I cannot prevent the infection itself.	1	2	3	4	5
Q12	Whether I get infected or not is a matter of luck.	1	2	3	4	5
Q13	What will happen is about to happen.	1	2	3	4	5

[Preventive Behaviors]

#	Practices of preventive behavior	Never	Sometime	Often	Always
	**In the last week, how often did you practice the followings?**				
Q14	Wore facial masks	1	2	3	4
Q15	Washed hands	1	2	3	4
Q16	Covered mouth with sleeve when coughing	1	2	3	4
Q17	Avoided visiting public places	1	2	3	4

Q18	Reduced the use of public transportation	1	2	3	4
Q19	Postponed or canceled social events	1	2	3	4
Q20	Refrained from going out	1	2	3	4
Q21	Stayed at home for three to four days if sick	1	2	3	4
Q22	Kept a 2 m distance from other people	1	2	3	4

[Backgrounds]

B1. What gender do you identify as?

1. Male
2. Female

B2. What is your age?

( ) years

B3. What is the highest degree or level of school you have completed?

1. Middle school
2. High school
3. Some college education
4. Bachelor’s degree
5. Some graduate education
6. Master’s degree, professional degree or higher

B4. Do you have any of the following diseases that were diagnosed or treated by a physician in the past year? Please select all.

1. Hypertension
2. Dyslipidemia
3. Stroke

4. Myocardial infarction, angina pectoris
5. Osteoarthritis, rheumatoid arthritis, osteoporosis
6. Pulmonary Tuberculosis
7. Asthma
8. Diabetes
9. Cancer

B5. How would you rate your health status?

1. Very bad
2. Bad
3. Normal
4. Good
5. Very good

B6. Which of these describes your monthly household income last year?

1. Less than 200 million KRW
2. 200 million to 399 million KRW
3. 400 million to 599 million KRW
4. 600 million KRW or above

B7. Which of these describes your current status of employment?

1. Unemployed
2. Employed
